# Autism-Associated Gene Expression in Peripheral Leucocytes Commonly Observed between Subjects with Autism and Healthy Women Having Autistic Children

**DOI:** 10.1371/journal.pone.0024723

**Published:** 2011-09-15

**Authors:** Yuki Kuwano, Yoko Kamio, Tomoko Kawai, Sakurako Katsuura, Naoko Inada, Akiko Takaki, Kazuhito Rokutan

**Affiliations:** 1 Department of Stress Science, Institute of Health Biosciences, The University of Tokushima Graduate School, Tokushima, Japan; 2 Department of Child and Adolescent Mental Health, National Institute of Psychiatry, National Center of Neurology and Psychiatry, Tokyo, Japan; 3 Chichibu-Gakuen Institution and Clinic for Intellectually Disabled Children, National Rehabilitation Center for Persons with Disabilities, Saitama, Japan; Wayne State University, United States of America

## Abstract

Autism spectrum disorder (ASD) is a severe neuropsychiatric disorder which has complex pathobiology with profound influences of genetic factors in its development. Although the numerous autism susceptible genes were identified, the etiology of autism is not fully explained. Using DNA microarray, we examined gene expression profiling in peripheral blood from 21 individuals in each of the four groups; young adults with ASD, age- and gender-matched healthy subjects (ASD control), healthy mothers having children with ASD (asdMO), and asdMO control. There was no blood relationship between ASD and asdMO. Comparing the ASD group with control, 19 genes were found to be significantly changed. These genes were mainly involved in cell morphology, cellular assembly and organization, and nerve system development and function. In addition, the asdMO group possessed a unique gene expression signature shown as significant alterations of protein synthesis despite of their nonautistic diagnostic status. Moreover, an ASD-associated gene expression signature was commonly observed in both individuals with ASD and asdMO. This unique gene expression profiling detected in peripheral leukocytes from affected subjects with ASD and unaffected mothers having ASD children suggest that a genetic predisposition to ASD may be detectable even in peripheral cells. Altered expression of several autism candidate genes such as *FMR-1* and *MECP2*, could be detected in leukocytes. Taken together, these findings suggest that the ASD-associated genes identified in leukocytes are informative to explore the genetic, epigenetic, and environmental background of ASD and might become potential tools to assess the crucial factors related to the clinical onset of the disorder.

## Introduction

Autism spectrum disorder (ASD) is a neurodevelopmental disorder characterized by impairments in reciprocal social interaction and communication, and restricted and repetitive behaviors and interests. Several twin studies have consistently demonstrated a higher concordance for monozygotic than dizygotic twins [1], [2], [3], [4]. Family studies have shown a marked increase in the occurrence rate of autism and milder phenotypes with subtle communication/social impairments or stereotypic behaviors among the relatives of autistic individuals compared with those of controls [5], [6]. Considering such evidence for familial aggregation of autism phenotypes with various degrees, multiple susceptibility factors, including genetic factors, are considered to be involved in the clinical manifestation of autism spectrum, interacting in complex ways with experiential factors during developmental courses [7].

The susceptibility genes to ASD remain largely unknown yet; however, recent molecular genomic approaches suggest that abnormal synaptic homeostasis represents a risk factor for ASD. For example, ASD-associated genes include *glutamate receptors*, *gamma-aminobutyric acid* (*GABA*) *receptors*, *neuroligins* (*NLGNs*), *neurexin 1*, *SH3 and multiple ankyrin repeat domains 3* (*SHANK3*), which are involved in synapse formation and function [8], [9], [10], [11], [12]. Several genes that are essential for neurological development, such as *roundabout, axon guidance receptor, homolog 1* (*ROBO1*) and *early growth response 2* (*EGR2*) are also differentially expressed in the lympnoblastoid cell lines from monozygotic twins discordant in severity of autism spectrum [13]. Neuronal differentiation- and survival-associated genes, such as *wingless-type MMTV integration site family member 2* (*WNT2*) and *homeobox A1* (*HOXA1*) were also reported as autism susceptibility genes [14], [15]. A large-scale single nucleotide polymorphism (SNP) association analysis involving 1,168 multiplex families revealed a single region on chromosome 11p12-p13 that exceeded the threshold for suggestive linkage as autism risk loci [16]. Recently, analysis in human postmortem cortical tissues from ASD cases has shown the association between a SNP in the promoter of the *met proto-oncogene* (*MET*) gene and a risk for ASD [17], [18]. This gene encodes the MET receptor tyrosine kinase and contributes to development of the cerebral cortex and cerebellum, which process may be disrupted in autism. Voineagu *et. al.* have reported that analysis of postmortem brain tissue samples from autism cases showed the splicing dysregulation and altered gene expression involved in neuronal and synaptic signaling dysfunction in the ASD brain [19].

Multiple susceptible factors are likely to influence directly or indirectly gene expression, resulting in functional alterations in proteins regulating synaptic homeostasis. The altered gene expression could be detected as gene expression signatures unique to ASD even in peripheral tissues. Increasing molecular genomic studies have indicated the importance of differential expression of ASD-associated genes in peripheral tissues as well as postmortem brains from subjects with ASD [13], [20], [21]. For example, gene expression profiling of lymphoblastoid cell lines are well-studied because of their characteristically homogeneity. These findings led us to speculate that ASD-associated changes in gene expression could be detectable in peripheral blood leucocytes from individuals with ASD.

In addition, identification of genetic etiology of ASD is hampered by the heterogeneity of autism phenotypes. One way of explaining this heterogeneity is broadening studies beyond the strict diagnosis of autism, by doing this, it may determine which components of the autistic symptoms are genetically transmitted and how these components interact [7]. To prove this hypothesis, we examined gene expression in peripheral blood from subjects with ASD and healthy mothers having children with ASD, and found an ASD-associated gene expression signature commonly observed in both individuals with ASD and healthy mothers having children with ASD, between whom there was no blood relationship. These findings may provide novel information for understanding the genetic background of ASD.

## Methods

### Study Subjects

The present study was approved by the Ethics Committee of the National Center of Neurology and Psychiatry (NCNP), Japan and by the Human Study Committee of the Tokushima University Hospital, Japan. After the experimental procedures were fully explained, written informed consent was obtained from each parent of minor subject and participants himself/herself, or from each adult subject. Adolescents and adults with ASD (17 males and 4 females; aged 26.7±5.5 y (mean±SD), range 18–38 y, n = 21) and the healthy women who had children with ASD (asdMO; 44.7±6.7 y, range 33–58 y, n = 21) were recruited from our research volunteer pool or specialized clinics and enrolled as the ASD group and the asdMO group, respectively. Subjects of the ASD group and those of asdMO group were biologically unrelated each other. The pre-existing clinical diagnoses of ASD were confirmed according to the Diagnostic and Statistical Manual of Mental Disorders (DSM-IV-TR, American Psychiatric Association, 2000) based on clinical interviews with subjects and/or parents using semi-structured interviews that were validated for Japanese pervasive developmental disorders (PDD) populations [22] by our research team including experienced child psychiatrist and developmental pediatrician. To corroborate the ASD diagnosis, the Japanese version of the Autism Spectrum Quotient (AQ-J) [23], [24] were completed by themselves and those scores except 3 subjects were above the cut-off of 26 (Table 1). Intellectual functions of the ASD subjects were evaluated using Japanese versions of WAIS-III: all ASD subjects exhibited normal IQ (mean full scale IQ; 91.86±21.62) (Table 1).

**Table 1:** Clinical characteristics of the ASD and asdMO groups.

ID	gender	age	AQ score	WAIS		
ASD (n = 21)			SUM	VIQ	PIQ	FIQ
1	M	18	34	126	109	122
2	M	18	30	91	92	91
3	M	20	.	67	82	71
4	M	20	32	88	61	72
5	M	22	31	70	54	60
6	M	23	.	59	62	54
7	M	24	30	109	95	104
8	M	24	31	106	91	100
9	M	26	36	88	70	78
10	M	26	30	72	75	71
11	M	27	31	103	84	94
12	M	28	28	98	64	82
13	M	29	.	77	83	78
14	M	29	32	114	128	121
15	M	32	24	132	115	127
16	M	34	21	108	106	107
17	M	38	29	92	64	78
18	F	27	19	111	102	108
19	F	28	37	85	78	80
20	F	32	30	119	110	117
21	F	35	39	105	112	114
mean±SD		26.67±5.53 y	30.22±5.06	96.19±19.92	87.48±21.62	91.86±21.62
asdMO (n = 21)						
1	F	33	.	.	.	.
2	F	36	.	.	.	.
3	F	37	.	.	.	.
4	F	39	.	.	.	.
5	F	39	.	.	.	.
6	F	41	.	.	.	.
7	F	41	.	.	.	.
8	F	41	.	.	.	.
9	F	42	.	.	.	.
10	F	44	.	.	.	.
11	F	46	.	.	.	.
12	F	46	.	.	.	.
13	F	47	.	.	.	.
14	F	47	.	.	.	.
15	F	49	.	.	.	.
16	F	49	.	.	.	.
17	F	50	.	.	.	.
18	F	51	.	.	.	.
19	F	51	.	.	.	.
20	F	58	.	.	.	.
21	F	58	.	.	.	.
mean±SD		44.73±6.66 y				

IQ = Intelligence Quotients; VIQ = Verbal IQ; PIQ = Performance IQ; FIQ = Full IQ; AQ = Autism-Spectrum Quotient; M = Male; F = Female; y = year.

Additionally, age- and sex-matched healthy volunteers were recruited from students or staffs of the Faculty of Medicine, the University of Tokushima and NCNP, and the local communities, and enrolled as controls for the ASD group (Control group; aged 27.0±5.5 y, range 19–39 y, n = 21) or controls for asdMO group (ctrlMO group; aged 44.7±6.7 y, range 31–59 y, n = 21). We confirmed that they did not have any serious physical or mental disorders including ASD in the past and at present, the children of the ctrlMO group have not been diagnosed as ASD and were in good health by interviews. All control subjects had not been medicated at least for three months prior to the recruitment.

### RNA preparation, amplification, and hybridization

Venous blood (5 ml) was taken from each subject and immediately poured into PAXgene^TM^ blood RNA tubes (Qiagen, Hilden, Germany). Total RNA was purified using a PAXgene Blood RNA kit (Qiagen) according to the manufacture's protocol. Contaminated DNA was removed using a DNase kit (Qiagen). The quality of the purified RNA and its applicability for microarray analysis were assessed by the Agilent 2100 Bioanalyzer using a RNA 6000 Nano Labchip kit (Agilent Technologies, Palo Alto, CA, USA). High quality RNA with a RIN number above 8.0 was used for microarray analysis and quantitative real-time reverse transcription-PCR (qPCR). For microarray analysis, total RNA (400 ng) was used for amplification and labeling of complementary RNA (cRNA) using a Low RNA Input Linear Amplification Kit PLUS and a succinimide-containing fluorescent dye (Cy3)-CTP according to the manufacturer's protocol (Agilent). Cy3-labeled cRNA was applied to the whole human genome oligoDNA microarray (4×44 k; Agilent) following the manufacturer's protocol. Hybridization was performed at 65°C for 17 h. After washing, fluorescence intensity at each spot was measured using a G2565BA Microarray Scanner (Agilent). Table S1 summarizes the array design and profile of subjects that were analyzed in this study.

### Microarray analysis

Signal intensities of Cy3 were quantified and analyzed by subtracting backgrounds using Feature Extraction 10 software (Agilent). Raw data output was imported into Genespring GX10 (Agilent) and normalized each chip to the 75th percentile of all measurements (per chip normalization), and normalized each probe to the median expression of matched control intensity for that gene across all samples (per gene normalization). Following normalization, we filtered genes having fluorescence intensities higher than a cutoff value of 50 among all samples.

### Pathway and functional analyses

The data sets of differentially expressed genes between the ASD and Control group, and the asdMO and ctrlMO group were analyzed using Ingenuity Pathway Analysis (IPA) application (Ingenuity Systems, Mountain View, CA, USA). IPA was used to identify molecular and cellular processes, biological functions, and modules of functionally related genes modified by the genes identified as significantly differentially expressed. IPA utilizes the knowledge in the literature about biological interactions among genes and proteins. The probability of a relationship between each biological function and the identified genes was calculated by Fisher's exact tests. The level of significance was set at *p-*value of 0.05. A detailed description is given in the online repository (http://www.ingenuity.com).

### Quantitative real-time reverse transcription PCR (qPCR) analysis

qPCR was done to validate the results obtained by the oligoDNA microarray. Total RNA (400 ng) from each sample was reverse transcribed with oligo dT primer and SuperScript™ III (invitrogen, Carlsbad, CA, USA). PCR reaction was performed with synthesized cDNA using the ABI-PRISM 7500 sequence detection system and TaqMan Gene Expression Master Mix (Applied Biosystems) according to the manufacture's protocols. For measurement of quantitative gene expression levels, we purchased oligonucleotide primers and gene-specific TaqMan probes from Applied Biosystems (Table S2). *Glyceraldehyde-3-phosphate dehydrogenase* (*GAPDH*) and *histone deacetylase 1*(*HDAC1*) were used as endogenous quantity controls. Data were analyzed using SDS 2.3 software (Applied Biosystems). Quantity values were normalized by *GAPDH* or *HDAC1* mRNA levels. After the relative ratios of each mRNA expression level between the ASD and Control group, and the asdMO and ctrlMO group were calculated, the unpaired *t* tests were used to compare the relative ratios for each mRNA for two pairs.

## Results

### Altered gene expression in peripheral blood in the ASD group

First, we examined differentially expressed genes in peripheral leukocytes between 21 subjects with ASD and their age- and gender-matched healthy controls. Microarray measurement showed that 19,194 probes in total had fluorescence intensities higher than a cut-off value of 50 among all samples. Unpaired *t* test with Benjamini-Hochberg correction for multiple comparisons at the 0.05 false discovery rate (FDR) selected 9,784 probes that were differentially expressed between the ASD and Control groups. The raw and normalized values for all samples by microarray analysis were deposited in the Gene Expression Omnibus (GEO) database (accession number: GSE26415). When the fold-change criterion was set at > 2-fold in the mean expression level, 28 probes passed the criterion and corresponded to 19 annotated genes. Among them, 18 genes (*NOVA2*, *SLC22A18AS*, *C21orf58*, *LZTR2*, *RKHD1*, *BC018095*, *FAM124A*, *LHB*, *TAOK2*, *UBL4A*, *ITGA2B*, *PLCXD2*, *NRN1*, *KLHDC7A*, *MYOG*, *NUMBL*, *WDTC2*, and *SDK2*) were significantly up-regulated, while one gene (*UTS2*) was down-regulated in the ASD group. These transcripts are listed according to greatest fold change in expression in Table 2.

**Table 2:** Differentially expressed genes for the ASD group compared with the Control group.

Accession No.	GeneSymbol	Description	fold change	corrected p-value
Up-regulated gene				
NM_002516	NOVA2	neuro-oncological ventral antigen 2	2.34	8.23E-03
NM_007105	SLC22A18AS	solute carrier family 22 (organic cation transporter), member 18 antisense	2.29	4.06E-03
NM_199071	C21ORF58	chromosome 21 open reading frame 58	2.25	4.19E-03
BC009106	LZTR2 (SEC16B)	leucine zipper transcription regulator 2	2.22	7.49E-03
NM_203304	RKHD1 (MEX3D)	ring finger and KH domain containing 1	2.22	4.27E-03
BC018095	BC018095	hypothetical protein LOC441869	2.21	6.92E-03
NM_145019	FAM124A	family with sequence similarity 124A	2.14	4.07E-03
NM_000894	LHB	luteinizing hormone beta polypeptide	2.13	1.09E-02
NM_016151	TAOK2	TAO kinase 2	2.13	1.03E-02
NM_014235	UBL4A	ubiquitin-like 4A	2.12	9.31E-03
NM_000419	ITGA2B	integrin, alpha 2b (platelet glycoprotein IIb of IIb/IIIa complex, antigen CD41)	2.12	4.90E-04
NM_153268	PLCXD2	phosphatidylinositol-specific phospholipase C, X domain containing 2	2.12	7.29E-03
NM_016588	NRN1	neuritin 1	2.10	4.90E-03
NM_152375	KLHDC7A	kelch domain containing 7A	2.08	1.37E-02
NM_002479	MYOG	myogenin (myogenic factor 4)	2.07	1.06E-02
NM_004756	NUMBL	numb homolog (Drosophila)-like	2.03	1.19E-02
NM_015023	WDTC1	WD and tetratricopeptide repeats 1	2.03	1.27E-02
NM_019064	SDK2	Homo sapiens sidekick homolog 2 (chicken)	2.01	4.70E-03
Down-regulated gene				
NM_021995	UTS2	urotensin 2	−4.25	1.67E-03

*corrected *p*-value was calculated by unpaired *t* test with Benjamini-Hochberg correction for multiple comparisons at the 0.05 false discovery rate (FDR).

**Figure 1:**
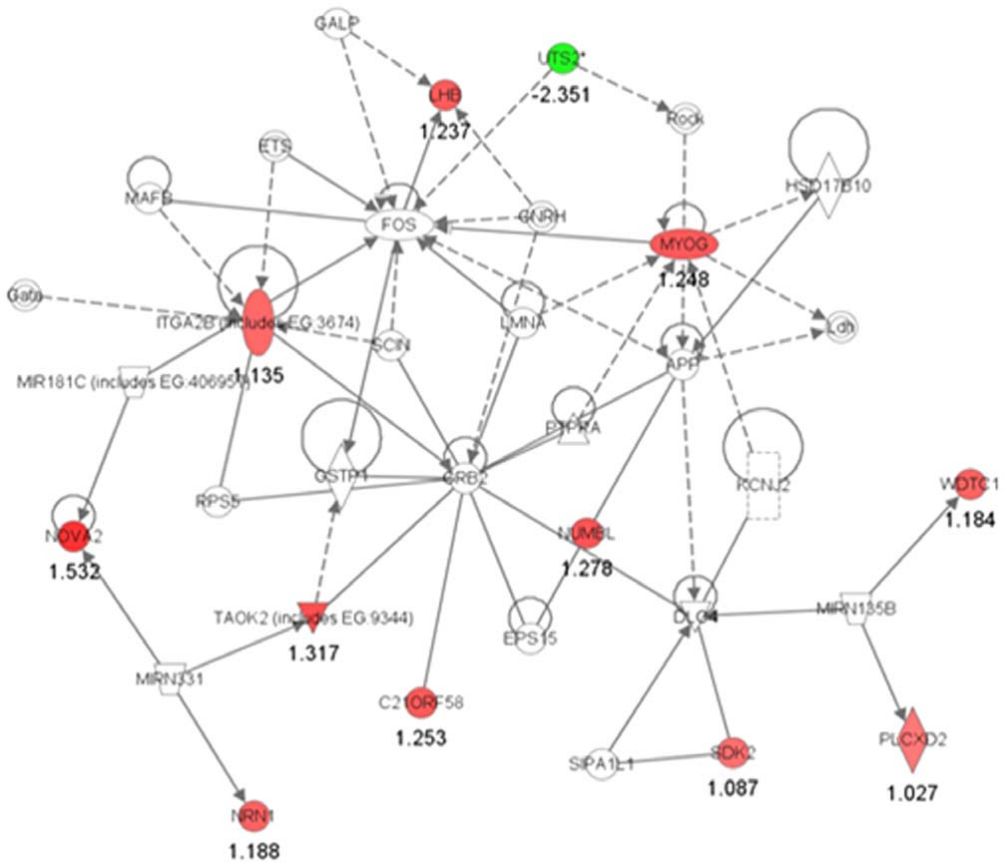
Ingenuity pathway analysis of differentially expressed genes for the ASD group compared with the Control group. The top scored network using the 19 differentially expressed genes for the ASD group (> 2-fold vs. control) is “cell morphology, cellular assembly and organization, nerve system development and function”, with 12 focus molecules and a score of 31. The network is displayed graphically as nodes (gene) and edges (the biological relationship between genes). The color intensity indicates the genes up-regulated or down-regulated in ASD are shown in red or green, respectively. The mean of log2 expression values compared with the Control group are described as logarithm scale.

The 19 differentially expressed genes were then subjected to the biofunctional pathway analysis using IPA. The net work analysis ranked “cell morphology, cellular assembly and organization, nerve system development and function” as the top-scoring net work (Figure 1). Particularly, 12 (*C21ORF58, ITGA2B, LHB, MYOG, NOVA2, NRN1, NUMBL, PLCXD2, SDK2, TAOK2, UTS2* and *WDTC1*) out of the 19 genes were included in this network. The molecules encoded by 11 up- or 1 down-regulated genes are shown in red and green in this network, respectively (Figure 1). The network analysis picked up several other networks, but only one or two genes were included in those networks. The top-scored biofunctions modified by the 19 genes significantly (*p*<0.05 by Fisher's exact test) were 1) cardiovascular disease (*p*-value: 3.46E-04), 2) vitamin and mineral metabolism (7.08E-04), 3) cell signaling (7.08E-04), 4) cellular development (9.90E-04), and 5) genetic disorder (9.90E-04). Although we examined the probability of a relationship between the identified 19 genes and each canonical pathway, IPA did not show any significantly modified pathway with *p* value of <0.01.

### Differentially expressed genes in the asdMO group

**Figure 2:**
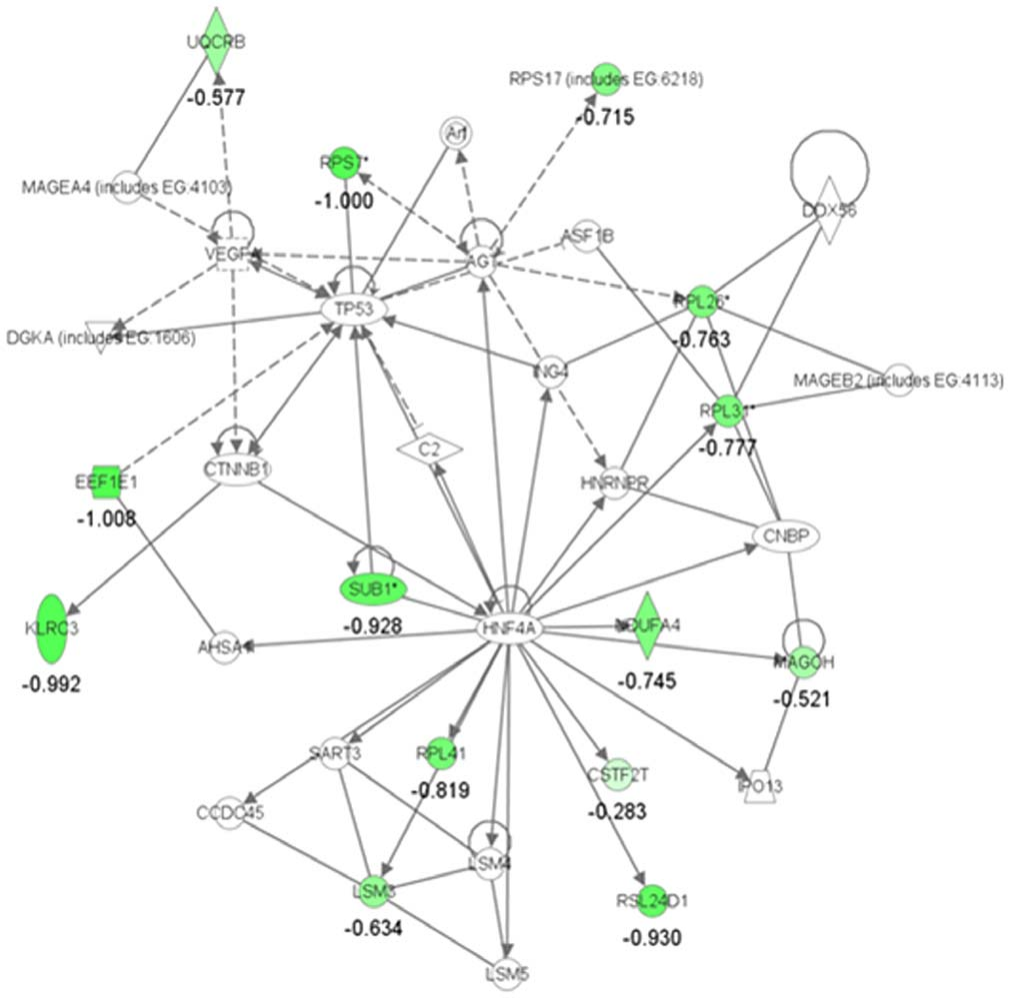
Ingenuity pathway analysis of differentially expressed genes for the asdMO group compared with the ctrlMO group. The top scored network using the 57 genes identified as differentially expressed genes for the asdMO group (> 2-fold vs. ctrlMO) is “cancer, RNA post-transcriptional modification, reproductive system disease”, with 14 focus molecules and a score of 27. The color intensity indicates the genes up-regulated or down-regulated in asdMO are shown in red or green, respectively. The mean of log2 expression values compared with the ctrlMO group are described as logarithm scale.

Using a similar way to that mentioned above for the ASD group, 57 annotated genes from 176 probes were identified to be differentially expressed in the asdMO group compared with the ctrlMO group (Table 3). The 57 genes were composed of 17 up-regulated and 40 down-regulated genes in the asdMO group. Analysis of the 57 genes with IPA indicated that “cancer, RNA post-transcriptional modification and reproductive system disease” was ranked as the top network in which 14 down-regulated genes were involved (Figure 2). The 57 genes significantly changed 57 biofunctional pathways in total. The most remarkable change was observed in the function of protein synthesis (*p*-value = 2.48E-08). Particularly, 10 genes encoding ribosomal proteins (*RPL7, RPL26, RPL31, RPL34, RPL39, RPL41, RPL9, RPS17, RPS27L*, and *RPS3A*) were significantly down-regulated in the asdMO group. Furthermore, several down-regulated (*CD69*, *TNFRSF17*, and *BCL2A1*) and up-regulated (*KRT1* and *C4B*) were involved in several immune functions, such as antigen presentation (*p*-value = 1.95E-07), cell-mediated immune response (*p*-value = 1.49E-05), and humoral immune response (*p*-value = 1.49E-05). As for the canonical pathway analysis, IPA did not reveal any significantly pathway modified by the 57 genes.

**Table 3:** Differentially expressed genes for the asdMO group compared with the ctrlMO group.

Accession No.	GeneSymbol	Description	fold change	corrected p-value
Up-regulated gene				
AK024445	C14ORF56	chromosome 14 open reading frame 56	2.85	1.16E-03
NM_020478	ANK1	ankyrin 1, erythrocytic	2.73	1.32E-03
NM_015431	TRIM58	tripartite motif-containing 58	2.66	3.81E-03
NM_138368	DKFZP761E198	DKFZp761E198 protein	2.54	2.40E-03
BC009106	LZTR2 (SEC16B)	SEC16 homolog B (S. cerevisiae)	2.33	4.54E-02
NM_000419	ITGA2B	integrin, alpha 2b (platelet glycoprotein IIb of IIb/IIIa complex, antigen CD41)	2.29	6.45E-03
NM_001266	CES1	carboxylesterase 1 (monocyte/macrophage serine esterase 1)	2.23	5.81E-03
NM_001002029	C4B	complement component 4B (Chido blood group)	2.21	2.46E-02
NM_001001957	OR2W3	olfactory receptor, family 2, subfamily W, member 3	2.15	1.50E-02
NM_000894	LHB	luteinizing hormone beta polypeptide	2.15	4.37E-02
NM_006121	KRT1	keratin 1	2.13	1.67E-02
NM_002501	NFIX	nuclear factor I/X (CCAAT-binding transcription factor)	2.09	3.31E-03
NM_001039476	NPRL3 (C16ORF35)	nitrogen permease regulator-like 3 (S. cerevisiae)	2.06	4.84E-03
NM_006798	UGT2A1	UDP glucuronosyltransferase 2 family, polypeptide A1	2.06	2.74E-02
NM_001024858	SPTB	spectrin, beta, erythrocytic	2.05	4.05E-03
NM_198149	SHISA4	shisa homolog 4 (Xenopus laevis)	2.02	1.20E-02
NM_007371	BRD3	bromodomain containing 3	2.02	1.11E-03
Down-regulated gene				
NM_002624	PFDN5	prefoldin subunit 5	−2.00	7.37E-03
NM_012459	TIMM8B	translocase of inner mitochondrial membrane 8 homolog B (yeast)	−2.01	8.93E-04
NM_014463	LSM3	LSM3 homolog, U6 small nuclear RNA associated (S. cerevisiae)	−2.01	1.59E-03
AB075859	ZNF525	zinc finger protein 525	−2.01	2.31E-03
NM_002489	NDUFA4	NADH dehydrogenase (ubiquinone) 1 alpha subcomplex, 4, 9kDa	−2.02	2.77E-03
NM_001803	CD52	CD52 molecule	−2.03	2.58E-03
NM_001019	RPS15A	ribosomal protein S15a	−2.03	3.73E-03
NM_005034	POLR2K	polymerase (RNA) II (DNA directed) polypeptide K, 7.0kDa	−2.05	2.56E-03
NM_001021	RPS17	ribosomal protein S17	−2.06	4.70E-03
NM_021104	RPL41	ribosomal protein L41	−2.09	6.64E-03
NM_001781	CD69	CD69 molecule	−2.09	4.54E-03
NM_005340	HINT1	histidine triad nucleotide binding protein 1	−2.12	1.96E-03
NM_000971	RPL7	ribosomal protein L7	−2.12	4.29E-03
NM_007333	KLRC3	killer cell lectin-like receptor subfamily C, member 3	−2.12	6.86E-03
NM_016304	RSL24D1 (C15orf15)	ribosomal L24 domain containing 1	−2.12	5.56E-03
NM_203495	COMMD6	COMM domain containing 6	−2.15	5.13E-03
NM_015920	RPS27L	ribosomal protein S27-like	−2.17	2.01E-03
NM_019051	MRPL50	mitochondrial ribosomal protein L50	−2.18	1.16E-03
NM_001026	RPS24	ribosomal protein S24	−2.18	2.80E-03
NM_002370	MAGOH	mago-nashi homolog, proliferation-associated (Drosophila)	−2.19	5.86E-04
NM_001192	TNFRSF17	tumor necrosis factor receptor superfamily, member 17	−2.23	1.83E-02
NM_005213	CSTA	cystatin A (stefin A)	−2.23	1.70E-03
NM_002586	PBX2	pre-B-cell leukemia homeobox 2	−2.27	3.17E-03
NM_000987	RPL26	ribosomal protein L26	−2.27	6.54E-03
NM_003096	SNRPG	small nuclear ribonucleoprotein polypeptide G	−2.30	2.26E-03
NM_004049	BCL2A1	BCL2-related protein A1	−2.32	4.72E-03
NM_001000	RPL39	ribosomal protein L39	−2.34	2.94E-03
NM_006294	UQCRB	ubiquinol-cytochrome c reductase binding protein	−2.34	2.79E-03
NM_000985	RPL17	ribosomal protein L17	−2.37	1.61E-03
NM_016093	RPL26L1	ribosomal protein L26-like 1	−2.37	2.28E-03
NM_015235	CSTF2T	cleavage stimulation factor, 3′ pre-RNA, subunit 2, 64kDa, tau variant	−2.41	1.80E-03
NM_000661	RPL9	ribosomal protein L9	−2.47	1.82E-03
NM_004280	EEF1E1	eukaryotic translation elongation factor 1 epsilon 1	−2.49	8.51E-04
NM_005127	CLEC2B	C-type lectin domain family 2, member B	−2.53	1.45E-03
NM_000993	RPL31	ribosomal protein L31	−2.57	3.59E-03
NM_006713	SUB1	SUB1 homolog (S. cerevisiae)	−2.59	2.15E-03
NM_001011	RPS7	ribosomal protein S7	−2.59	1.70E-03
NM_033625	RPL34	ribosomal protein L34	−2.64	3.73E-03
BC049823	RPL22L1	ribosomal protein L22-like 1	−2.84	1.16E-03
NM_001006	RPS3A	ribosomal protein S3A	−3.08	1.64E-03

*corrected *p*-value was calculated by unpaired *t* test with Benjamini-Hochberg correction for multiple comparisons at the 0.05 FDR.

### Similarity in gene expression profiling between the ASD and asdMO group

**Figure 3:**
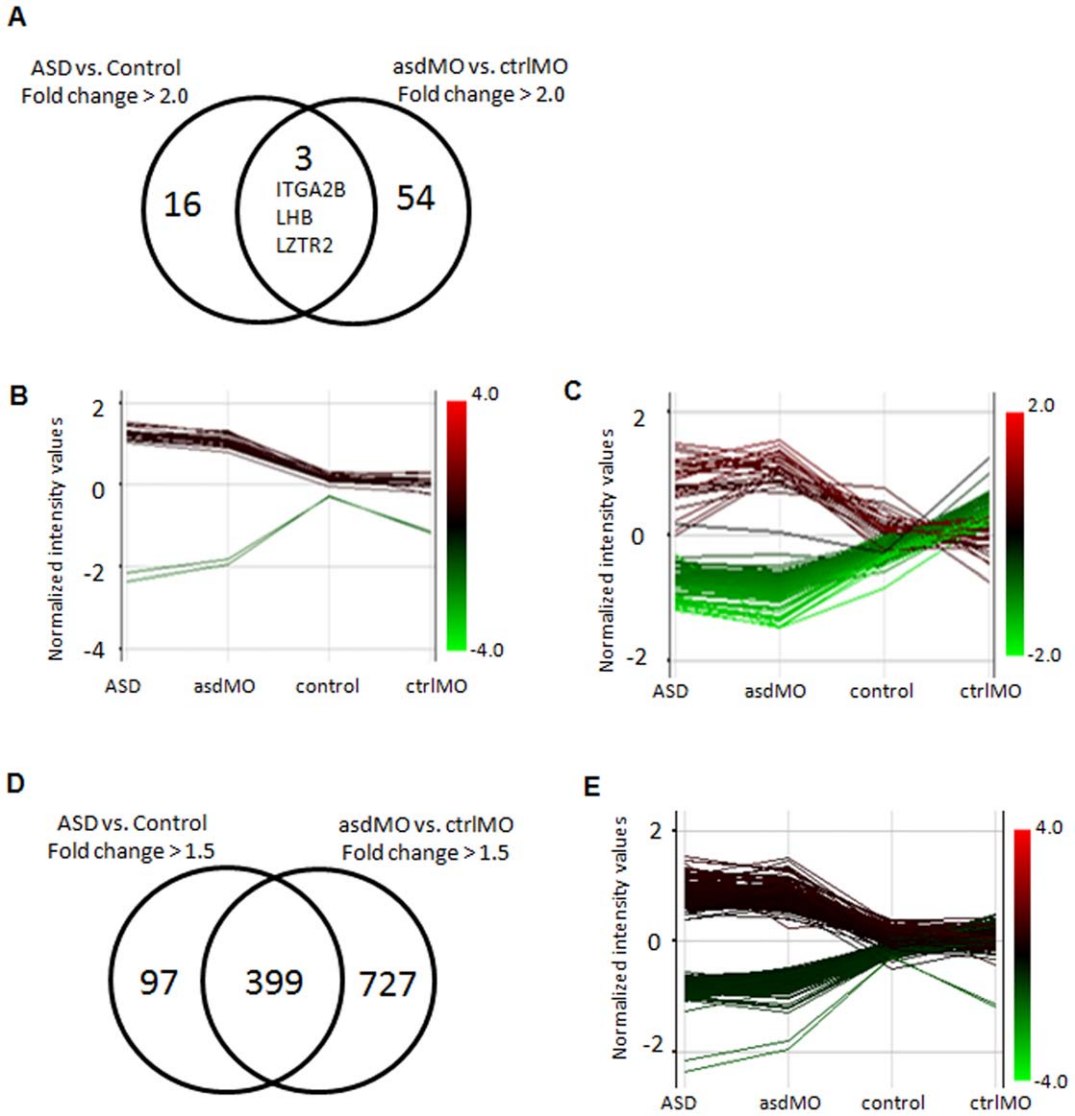
Similarity in gene expression profiling between the ASD and asdMO group. Venn diagram of differentially expressed genes for the ASD group compared with the Control group and the asdMO group compared with the ctrlMO group (> 2-fold change) (A). RNA was prepared from each group and subjected to microarray analysis. Differentially expressed 19 and 57 genes by >2-fold in ASD group and asdMO group, respectively. Three genes were overlapped. The mean of expression values of the 19 genes (B) and the 57 genes (C) changed in a parallel direction between the ASD and asdMO group. The normalized intensity value of genes, which was up-regulated or down-regulated by 2-fold, is shown in red or green, respectively, compared with control or ctrlMO. Venn diagram of differentially expressed 496 and 1126 genes by >1.5-fold in ASD group and asdMO group (D). 399 genes were overlapped. The mean of expression values of the overlapped 399 genes was similar direction between the ASD and asdMO group (E). The normalized intensity value of genes, which was up-regulated or down-regulated by 1.5-fold, is shown in red or green, compared with control.

As described above, we identified 19 differentially expressed genes in young subjects with ASD compared their age- and sex-matched controls, and 57 differentially expressed genes comparing healthy women with and without ASD children. Among the 19 and 57 genes which were separately identified according to our 2-fold change criterion by two comparisons, three genes (*ITGA2B*, *LHB*, and *LZTR2*) overlapped (Figure 3A). It should be noted that expression of the 19 genes (Figure 3B) and the 57 genes (Figure 3C) changed in a parallel direction between the ASD and asdMO group, indicating that healthy women having ASD children shared some gene expression signature in common with individuals diagnosed as ASD, although they had no full symptoms above clinical threshold of ASD. When the fold change criterion was lowered from > 2.0 to > 1.5, 496 differentially expressed genes were identified in the ASD group compared with the Control group. In this case, 399 out of 496 genes were also included in 1126 genes differentially expressed in the asdMO group compared with the ctrlMO group by a looser cutoff of 1.5-fold change (Figure 3D), and the altered direction of 399 genes was similar between the ASD and asdMO group (Figure 3E). The 399 overlapping genes are listed in Table S3. We also examined a pathway or functional analysis of the 399 genes. As shown in Table 4, the network analysis ranked “Nervous System Development and Function, Tissue Development, Genetic Disorder” as the top-scoring net work. Subsequently, the overlapping gene set modified “Cell-To-Cell Signaling and Interaction, Tissue Development, Embryonic Development” and “Gene Expression, Cellular Growth and Proliferation, Hematological System Development and Function”. The top-scored biofunctions modified by the overlapping genes were 1) Cell-To-Cell Signaling and Interaction (*p*-value: 5.86E-06), 2) Reproductive System Development and Function (5.86E-06), 3) Tissue Development (5.86E-06). These results indicated that some genetic predisposition of ASD specifically found in the ASD group was also shared by only healthy women having ASD children, but not healthy women having non-ASD children or healthy adults, which could be detectable as an ASD-associated gene expression signature in peripheral leukocytes.

**Table 4:** Biological roles of overlapping genes (>1.5-fold change) between ASD and asdMO group.

*A) Associated Network Functions for overlapping genes*		
	score	
Nervous System Development and Function, Tissue Development, Genetic Disorder	40	
Cell-To-Cell Signaling and Interaction, Tissue Development, Embryonic Development	36	
Gene Expression, Cellular Growth and Proliferation, Hematological System Development and Function	34	

*B) Top Biofunctions for overlapping genes*		
	*P-value* range	No. of molecules
Cell-To-Cell Signaling and Interaction	5.86E-06 - 2.41E-02	44
Reproductive System Development and Function	5.86E-06 - 2.19E-02	19
Tissue Development	5.86E-06 - 2.69E-02	30
Hematological Disease	1.04E-05 - 2.46E-02	24
Gastrointestinal Disease	4.65E-05 - 2.45E-02	13

### Confirmation by qPCR

Based on the microarray data, we selected putative ASD-associated genes (16 out of the 19 ASD-associated genes identified by comparison between the ASD and Control group, and 19 out of 57 asdMO-associated genes by comparison between the asdMO and ctrlMO group, shown in Table 5) and validated changes in their expression levels using qPCR. Among the 19 ASD-associated genes, three mRNA levels (*C21orf58*, *SDK2*, and *BC018095*) could not be measured, since appropriate TaqMan gene probes for them were not available. Among the 57 asdMO-associated genes, we preferentially selected a group of genes encoding key molecules in the regulation of the top-scoring biological function (protein synthesis). *GAPDH* mRNA levels were used as an internal control. In addition, we measured *HDAC1* mRNA levels as another internal quantity control, since the microarray measurement showed constant expression levels of these two mRNAs among all subjects, which were also confirmed by qPCR. The result using *GAPDH* as an internal control was similar to that using *HDAC1* instead (data not shown).

**Table 5:** Real-time qPCR validation of ASD-related/asdMO-related gene expression levels (relative mRNA expression to *GAPDH*).

	Control	ASD	ctrlMO	asdMO	*p*-value	
Gene	relative expression	expression value	expression value	expression value		
	mean±SEM	mean±SEM	mean±SEM	mean±SEM	ASD vs control	asdMO vs ctrlMO
(ASD-related)						
ITGA2B	4.68±0.92	8.47±0.94	5.62±2.35	9.64±1.93	4.0E-03*	5.11E-03*
NRN1	3.53±0.40	5.41±0.59	3.92±0.58	6.65±0.85	2.2E-02*	4.08E-02*
WDTC1	1.23±0.09	1.57±0.11	1.27±0.10	1.60±0.16	2.5E-02*	3.50E-01
PLCXD2	1.09±0.11	0.73±0.06	0.93±0.09	0.79±0.11	6.2E-03*	6.01E-02
UTS2	1.24±0.31	1.00±0.30	0.98±0.21	0.61±0.16	8.1E-02	4.55E-01
KLHDC7A	1.17±0.17	0.80±0.10	1.00±0.20	0.78±0.23	6.0E-01	4.89E-01
LHB	2.03±0.21	1.74±0.19	2.21±0.37	1.81±0.24	3.5E-01	3.82E-01
LZTR2	0.68±0.68	0.55±0.54	1.03±1.03	0.76±0.76	6.3E-01	5.88E-01
NUMBL	0.72±0.07	0.90±0.14	1.17±0.21	1.17±0.29	8.1E-01	7.39E-01
RKHD1	1.02±0.14	0.84±0.14	1.36±0.18	1.10±0.21	2.6E-01	1.38E-01
SLC22A18AS	4.53±0.49	4.65±0.46	3.79±0.40	5.32±0.84	6.9E-01	3.67E-01
TAOK2	0.62±0.06	0.78±0.11	0.93±0.14	0.87±0.19	2.1E-01	3.84E-01
UBL4A	1.06±0.06	1.15±0.08	1.04±0.05	1.02±0.11	3.6E-01	4.46E-01
NOVA2	u.d.	u.d.	u.d.	u.d.		
FAM124A	u.d.	u.d.	u.d.	u.d.		
MYOG	u.d.	u.d.	u.d.	u.d.		
(asdMO-related)						
CSTA	0.55±0.06	0.40±0.05	0.81±0.11	0.39±0.07	2.9E-02*	3.45E-04*
CLEC2B	0.88±0.06	0.68±0.12	1.50±0.14	0.88±0.13	1.5E-02*	7.50E-04*
RPL34	0.71±0.06	0.51±0.10	1.07±0.13	0.63±0.12	8.8E-03*	2.04E-03*
EEF1E1	0.49±0.05	0.34±0.06	0.58±0.05	0.38±0.07	5.5E-03*	4.39E-03*
RPL9	0.50±0.07	0.34±0.08	0.68±0.09	0.42±0.12	1.0E-02*	4.85E-03*
RPS3A	0.51±0.05	0.35±0.06	0.64±0.07	0.40±0.09	3.7E-03*	3.31E-03*
CES1	2.56±0.41	10.09±4.63	2.60±0.48	5.56±0.89	5.4E-03*	6.88E-03*
ANK1	7.98±1.32	12.23±1.44	7.80±1.37	18.29±2.87	2.3E-02*	5.88E-03*
BCL2A1	0.69±0.10	0.51±0.10	1.28±0.30	0.65±0.12	1.1E-01	1.26E-02*
RPS7	0.56±0.07	0.29±0.06	0.74±0.14	0.37±0.07	2.5E-03*	7.73E-03*
SUB1	0.32±0.04	0.21±0.04	0.36±0.04	0.28±0.07	4.9E-03*	3.06E-02*
TRIM58	5.92±0.77	9.71±1.11	6.42±1.37	12.41±2.36	1.4E-02*	2.51E-02*
RPL39	0.72±0.17	0.38±0.09	0.77±0.13	0.52±0.14	2.4E-02*	3.18E-02*
SPTB	2.93±0.45	4.89±0.66	3.32±0.78	8.02±1.78	2.2E-02*	2.98E-02*
UQCRB	0.49±0.05	0.41±0.05	0.51±0.05	0.39±0.03	1.5E-01	1.98E-01
PBX2	0.92±0.09	0.94±0.12	0.74±0.14	1.14±0.23	8.4E-01	6.46E-02
SNRPG	0.55±0.06	0.40±0.06	0.53±0.07	0.42±0.06	1.8E-02*	2.75E-01
NDUFA4	2.81±0.37	2.89±0.38	2.68±0.36	3.36±0.39	7.4E-01	9.06E-02
(putativeASD-related)						
FMR1	1.05±0.06	0.71±0.07	1.00±0.12	0.92±0.10	3.8E-03*	7.13E-01
MECP2	1.01±0.04	1.22±0.08	0.99±0.05	1.33±0.09	4.5E-02*	3.47E-03*
SLC9A6	1.74±0.15	1.37±0.13	1.53±0.15	1.62±0.17	6.4E-02	4.88E-01
UBE3A	0.93±0.05	0.88±0.09	0.81±0.04	0.88±0.08	3.1E-01	6.50E-01
MET	u.d.	u.d.	u.d.	u.d.		
NRXN1	u.d.	u.d.	u.d.	u.d.		

*p*-value was calculated by *t* test using control (n = 21) and ASDs (n = 21), and ctrlASD (n = 21) and asdMO (n = 19).

*p<0.05.

Of the 16 ASD-associated genes, we could confirm significant changes in mRNA levels in only four genes (*ITGA2B*, *NRN1*, *WDTC1*, and *PLCXD2*). *ITGA2B* and *NRN1* were also detected as significantly up-regulated in the asdMO group (Table 5). Changes in the other mRNA levels were not significant.

Of the measured 19 asdMO-associated genes, 14 genes (*CSTA, CLEC2B, RPL34, EEFE1*, *RPL9, RPS3A, CES1, ANK1, BCL2A1, RPS7, SUB1, TRIM58, RPL39,* and *SPTB*) were validated to significantly alter their mRNA levels. All genes except *BCL2A1* also significantly changed their expression in the same direction in the ASD group (Table 5).

In addition to the 35 genes described above, we measured mRNA levels of six genes known as putative ASD-related genes based on the previous studies; *FMR1*, *MET*, *MECP2*, *NRXN1*, *UBE3A*, and *SLC9A6* [25]. All these genes were not included in the 19 ASD-associated genes when identified using the 2-fold criterion, but *MECP2*, *NRXN1*, *UBE3A*, and *SLC9A6* were included in the 496 ASD-associated genes, when the fold-change criterion was set at >1.5-fold. As shown in Table 5, *MECP2* mRNA levels were found to elevate significantly in both the ASD and asdMO groups by qPCR, and *FMR1* expression significantly reduced only in the ASD group, compared with the respective controls.

## Discussion

Using a whole human genome microarray and the statistical analysis with 2-fold change criteria, we first identified the 19 genes differentially expressed in peripheral leukocytes between subjects with ASD and their age- and sex-matched controls. These ASD-associated genes were preferentially included in the network of cell morphology, cellular assembly and organization, nerve system development and function. In addition, we identified the 57 genes whose mRNA levels were significantly different between unaffected mothers having children with ASD and mothers having children without any mental disorders. Differential expression of the 57 genes could be characterized as significant alterations of biosynthesis of protein and processing of ribosomal RNA. Intriguingly, unaffected mothers having children diagnosed with ASD possessed a unique gene expression signature, which could also be detected in the affected subjects with ASD, although the mothers having affected children and affected subjects had no direct heredity of blood between them.

Based on the twin and family studies, the etiology of autism has a substantial genetic component [25]. With genome-wide differential display approaches, a number of recent studies have highlighted SNPs, copy number variants (CNVs), and epigenetic factors in the dysregulated expression of candidate genes related to the occurrence of autism [26]. Putative and known candidates for susceptibility to autism are categorized to differentiation of neurons (*e.g. DISC1*, *MET*, *PTEN*, and *ITGB3*), neuronal cell adhesion (*e.g. NRXN1*, *NLGN3*, and *NLGN4X*), transmission of nervous system (*e.g. OXTR*, *SLC6A4*, *GABRB3*, and *SHANK3*), and regulation of neuronal activity (*e.g. FMR1*, *MECP2*, and *UBE3A*).

In addition to the known ASD-related genes (*FMR1*, *MET*, *MECP2*, *NRXN1*, *UBE3A*, and *SLC9A6*), we selected 36 genes from the 19 and 57 genes, and then validated changes in mRNA levels of 42 genes in total by qPCR. Finally, we found that expression of the 15 genes (*ITGA2B*, *NRN1*, *CSTA, CLEC2B, RPL34, EEFE1*, *RPL9, RPS3A, CES1, ANK1, RPS7, SUB1, TRIM58, RPL39,* and *SPTB*) was significantly changed to the same direction in both ASD subjects and women having ASD children (Table 5).

We observed that mRNA levels of *ITGA2B* encoding glycoprotein (GP) αIIβ were up-regulated about 2-fold in the peripheral leukocytes of the both ASD and asdMO groups (Table 5). GP αIIβ forms αIIbβ3 integrin with GP βIII encoded by *ITGB3* [27]. *ITGB3* is known as an autism-susceptible gene, which was identified as a male quantitative trait locus for whole blood serotonin levels [28]. αIIbβ3 integrin has an important role in the cell morphology, including synapse maturation. The increased expression of *ITGA2B* mRNA might modify cellular morphology of peripheral cells in mothers having children with ASD as well as subjects with ASD.

Another interesting observation was the reduction of *SUB1* mRNA expression in ASD and asdMO groups. *SUB1* encodes a transcription factor that activates RNA polymerase II transcription. A recent study has shown that *SUB1* is also involved in the transcription of the MET receptor tyrosine kinase [29]. The significant down-regulation of *SUB1* observed in our ASD subjects may be related to the reduction of MET protein reported in ASD cases [17], [18].

In addition to the important findings described above, validation of candidate genes by qPCR provided novel and unique gene expression changes in subjects with ASD and healthy women having ASD children. Interestingly, several genes encoding ribosomal proteins (*RPL34*, *RPL9*, *RPS3A*, *RPS7*, and *RPL39*) were all down-regulated in both ASD and asdMO groups. The ribosomal proteins play a crucial role in the regulation of protein synthesis; therefore, their expression must be strictly controlled [30]. Several lines of evidence have identified the linkage between ribosome biogenesis and diseases such as cancer, anemia, and aging. A recent review has also emphasized that protein synthesis is tightly linked to the regulation of neurological processes and cell growth [31]. The down-regulation of the genes encoding ribosomal proteins may indirectly reflect some atypical process of neurological development in subjects with ASD and also mothers having children with ASD.

qPCR also validated differential expression of two genes (*MECP2* and *fragile X mental retardation-1* (*FMR1*)), well-known candidates for susceptibility to autism, in ASD and/or asdMO group. The *MECP2* gene encodes the X-linked methyl CpG binding protein 2 and its mutations and dysfunctions have also been associated with a broad array of other neurodevelopmental disorders including autism [32]. Consistent with increased expression of *MECP2* in our study, several reports show that overexpression of *MECP2* by gene duplication is a cause of neurodevelopmental delay in human clinical cases [33], [34]. Mutation in the *MECP2* gene identical to some classical Rett syndrome mutations has been identified in small numbers of autistic phenotypes in females [35], [36]. Linkage analysis has identified the over- and undertransmittance of specific *MECP2* variants in families with autistic children [37]. Taken together, these studies suggested a link between *MECP2* and susceptibility to autism. The phenotypic differences between different clinical diagnoses may arise from differences in genetic background and environmental exposures in each affected individual. *MECP2* may serve as a central epigenetic regulator of activity-dependent synaptic maturation.

The mutation of *FMR1* triggers abnormal methylation, resulting in a loss of *FMR1* expression in the lymphoblastoid cells in Fragile X syndrome, and such loss of FMR1 expression was also reported in the frontal cortex of postmortem brains from subjects with ASD [38]. In parallel with these findings, our subjects with ASD, but not unaffected mothers having affected children, showed significantly decreased *FMR1* mRNA levels compared with their controls (Table 5). One might say that *FMR1* may be involved in overt manifestations of ASD.

Although the underlying mechanism of autism is considered to be closely associated with brain maldevelopment, our results mentioned above suggest that the dysregulated expression of multiple candidate genes could be at least in part detectable in peripheral leukocytes. In fact, Hu *et*. *al*. demonstrated that the ASD-associated changes in gene expression involving in nervous system development could be detected in lymphoblastoid cell lines [39], [40]. Recent studies have suggested the possibility of identifying blood biomarkers by gene expression profiling studies in patients with mood disorders [41] and psychosis [42]. Moreover, Alter *et*. *al*. have shown autism- and increased paternal age-related changes in global levels of gene expression regulation in peripheral blood lymphocytes [43]. Thus, several lines of evidence suggest that gene expression signatures for neurodevelopmental disorders may be detectable in peripheral blood leukocytes. More recently, Voineagu *et. al.* have reported that the expression levels of neuronal specific splicing factor *A2BP1* were dysregulated in the ASD brain [19]. The splicing of *A2BP1*-dependent alternative exons was also altered. However, in our cases, *A2BP1* mRNA expression was not significantly changed in peripheral blood lymphocytes.

The implications of our approach suggest that several genes commonly found in both affected subjects and unaffected mothers having affected children might be potentially useful for understanding of mechanisms shaping the pathophysiology of ASD. Among our validated candidates, decreased expression of *FMR1* [38] and increased expression of *MECP2* [33] were already reported in lymphoblastoid cells and peripheral blood lymphocytes, respectively. However, the other candidate genes such as *ITGA2B*, *SUB1*, and ribosomal protein-related genes have not been documented. Identifying such genes could be helpful for objective diagnosis not solely relied on overt behavioral manifestations. The unique signatures observed in this study are informative to explore the genetic, epigenetic, and environmental background of ASD and might be a potential tool to find out the crucial factors-related to the overthreshold and subthreshold manifestations of ASD. To confirm these novel findings, we need more evidence established by case-case analysis and animal- and molecular-based studies. To show the predictive ability of the identified marker genes, a completely independent cohort study with larger numbers of subjects is currently underway.

## Supporting Information

Table S1 **Experimental design for DNA microarrays**.
(DOC)

Table S2 **TaqMan probe ID or sequences of primers for qRT-PCR analysis.**
(DOC)

Table S3 **The list of overlapping genes between the ASD and asdMO group (fold-change > 1.5, vs. control or ctrlMO.**
(XLS)
