# Supplementary material for: Autism-Associated Gene Expression in Peripheral Leucocytes Commonly Observed between Subjects with Autism and Healthy Women Having Autistic Children
Source: PLoS One. 2011 Sep 15;6(9):e24723. doi: 10.1371/journal.pone.0024723 (PMC3174190; doi:10.1371/journal.pone.0024723)
Supplement: Table S1 — Experimental design for DNA microarrays. (DOC) [file pone.0024723.s001.doc]

| Table S1. Experimental design for DNA microarrays | | | | | |
| --- | --- | --- | --- | --- | --- |
|  |  |  |  |  |  |
|  | Individual ID# | Status | Age | Gender | Microarray ID# |
| 1 | 3842 | ASD | 18 | M | ASD3842 |
| 2 | 3757 | cont | 19 | M | ASD3757 |
| 3 | 3843 | ASD | 18 | M | ASD3843 |
| 4 | 3759 | cont | 19 | M | ASD3759 |
| 5 | 3623 | ASD | 20 | M | ASD3623 |
| 6 | 3723 | cont | 20 | M | ASD3723 |
| 7 | 3865 | ASD | 20 | M | ASD3865 |
| 8 | 3749 | cont | 20 | M | ASD3749 |
| 9 | 3617 | ASD | 22 | M | ASD3617 |
| 10 | 2833 | cont | 22 | M | ASD2833 |
| 11 | 3622 | ASD | 23 | M | ASD3622 |
| 12 | 2834 | cont | 23 | M | ASD2834 |
| 13 | 3326 | ASD | 24 | M | ASD3326 |
| 14 | 3795 | cont | 24 | M | ASD3795 |
| 15 | 3332 | ASD | 24 | M | ASD3332 |
| 16 | 3825 | cont | 25 | M | ASD3825 |
| 17 | 3844 | ASD | 26 | M | ASD3844 |
| 18 | 2799 | cont | 26 | M | ASD2799 |
| 19 | 3867 | ASD | 26 | M | ASD3867 |
| 20 | 3875 | cont | 26 | M | ASD3875 |
| 21 | 3616 | ASD | 27 | F | ASD3616 |
| 22 | 3876 | cont | 26 | F | ASD3876 |
| 23 | 3863 | ASD | 27 | M | ASD3863 |
| 24 | 3877 | cont | 28 | M | ASD3877 |
| 25 | 3866 | ASD | 28 | M | ASD3866 |
| 26 | 3878 | cont | 28 | M | ASD3878 |
| 27 | 3328 | ASD | 28 | F | ASD3328 |
| 28 | 3879 | cont | 29 | F | ASD3879 |
| 29 | 3624 | ASD | 29 | M | ASD3624 |
| 30 | 2800 | cont | 31 | M | ASD2800 |
| 31 | 3864 | ASD | 29 | M | ASD3864 |
| 32 | 2801 | cont | 31 | M | ASD2801 |
| 33 | 3831 | ASD | 32 | M | ASD3831 |
| 34 | 2871 | cont | 33 | M | ASD2871 |
| 35 | 3619 | ASD | 32 | F | ASD3619 |
| 36 | 1934 | cont | 30 | F | ASD1934 |
| 37 | 3331 | ASD | 34 | M | ASD3331 |
| 38 | 2872 | cont | 33 | M | ASD2872 |
| 39 | 3870 | ASD | 35 | F | ASD3870 |
| 40 | 3880 | cont | 34 | F | ASD3880 |
| 41 | 3829 | ASD | 38 | M | ASD3829 |
| 42 | 2836 | cont | 39 | M | ASD2836 |
| 43 | 3837 | asdMO | 33 | F | ASD3837 |
| 44 | 2689 | ctrlMO | 31 | F | ASD2689 |
| 45 | 3333 | asdMO | 36 | F | ASD3333 |
| 46 | 1922 | ctrlMO | 38 | F | ASD1922 |
| 47 | 3836 | asdMO | 37 | F | ASD3836 |
| 48 | 3873 | ctrlMO | 39 | F | ASD3873 |
| 49 | 3832 | asdMO | 39 | F | ASD3832 |
| 50 | 3626 | ctrlMO | 39 | F | ASD3626 |
| 51 | 3840 | asdMO | 39 | F | ASD3840 |
| 52 | 1491 | ctrlMO | 39 | F | ASD1491 |
| 53 | 3830 | asdMO | 41 | F | ASD3830 |
| 54 | 1405 | ctrlMO | 41 | F | ASD1405 |
| 55 | 3833 | asdMO | 41 | F | ASD3833 |
| 56 | 1415 | ctrlMO | 41 | F | ASD1415 |
| 57 | 3612 | asdMO | 41 | F | ASD3612 |
| 58 | 3872 | ctrlMO | 41 | F | ASD3872 |
| 59 | 3327 | asdMO | 42 | F | ASD3327 |
| 60 | 3871 | ctrlMO | 42 | F | ASD3871 |
| 61 | 3839 | asdMO | 44 | F | ASD3839 |
| 62 | 1416 | ctrlMO | 43 | F | ASD1416 |
| 63 | 3834 | asdMO | 46 | F | ASD3834 |
| 64 | 1574 | ctrlMO | 44 | F | ASD1574 |
| 65 | 3838 | asdMO | 46 | F | ASD3838 |
| 66 | 3334 | ctrlMO | 44 | F | ASD3334 |
| 67 | 3835 | asdMO | 47 | F | ASD3835 |
| 68 | 3335 | ctrlMO | 45 | F | ASD3335 |
| 69 | 3613 | asdMO | 47 | F | ASD3613 |
| 70 | 3874 | ctrlMO | 46 | F | ASD3874 |
| 71 | 3618 | asdMO | 49 | F | ASD3618 |
| 72 | 3868 | ctrlMO | 47 | F | ASD3868 |
| 73 | 3841 | asdMO | 49 | F | ASD3841 |
| 74 | 3869 | ctrlMO | 48 | F | ASD3869 |
| 75 | 3621 | asdMO | 50 | F | ASD3621 |
| 76 | 1387 | ctrlMO | 49 | F | ASD1387 |
| 77 | 3620 | asdMO | 51 | F | ASD3620 |
| 78 | 2859 | ctrlMO | 52 | F | ASD2859 |
| 79 | 3330 | asdMO | 51 | F | ASD3330 |
| 80 | 1490 | ctrlMO | 53 | F | ASD1490 |
| 81 | 3625 | asdMO | 58 | F | ASD3625 |
| 82 | 1467 | ctrlMO | 57 | F | ASD1467 |
| 83 | 3329 | asdMO | 58 | F | ASD3329 |
| 84 | 1816 | ctrlMO | 59 | F | ASD1816 |
