# Supplementary material for: Autism-Associated Gene Expression in Peripheral Leucocytes Commonly Observed between Subjects with Autism and Healthy Women Having Autistic Children
Source: PLoS One. 2011 Sep 15;6(9):e24723. doi: 10.1371/journal.pone.0024723 (PMC3174190; doi:10.1371/journal.pone.0024723)
Supplement: Table S2 — TaqMan probe ID or sequences of primers for qRT-PCR analysis. (DOC) [file pone.0024723.s002.doc]

Table S2. TaqMan probe ID or sequences of primers for qRT-PCR analysis

| Gene symbol | TaqMan probe ID / PCR primers |  |
| --- | --- | --- |
| ITGA2B | Hs01116228_m1 |  |
| WDTC1 | Hs00248234_m1 |  |
| PLCXD2 | Hs00327047_m1 |  |
| UTS2 | Hs00254354_m1 |  |
| KLHDC7A | Hs00537061_s1 |  |
| LHB | Hs00751207_s1 |  |
| LXTR2 | Hs00263939_m1 |  |
| NUMBL | Hs00191080_m1 |  |
| RKHD1 | Hs00418289_m1 |  |
| SLC22A18AS | Hs00757934_m1 |  |
| TAOK2 | Hs00191170_m1 |  |
| UBL4A | Hs00204501_m1 |  |
| NOVA2 | Hs00159636_m1 |  |
| FAM124A | Hs00290069_m1 |  |
| MYOG | Hs01072232_m1 |  |
| CSTA | Hs00193257_m1 |  |
| CLEC2B | Hs00192860_m1 |  |
| RPL34 | Hs00241560_m1 |  |
| EEF1E1 | Hs00188710_m1 |  |
| RPL9 | Hs01552541_g1 |  |
| RPS3A | Hs00832893_sH |  |
| CES1 | Hs00275607_m1 |  |
| ANK1 | Hs00252833_m1 |  |
| BCL2A1 | Hs00187845_m1 |  |
| RPS7 | Hs00361391_g1 |  |
| SUB1 | Hs00743451_s1 |  |
| TRIM58 | Hs00296057_m1 |  |
| RPL39 | Hs02598234_g1 |  |
| SPTB | Hs01024103_m1 |  |
| UQCRB | Hs01890823_s1 |  |
| PBX2 | Hs00855025_s1 |  |
| SNRPG | Hs01923241_g1 |  |
| NDUFA4 | Hs00800172_s1 |  |
| FMR1 | Hs00924547_m1 |  |
| MECP2 | Hs00172845_m1 |  |
| SLC9A6 | Hs00234723_m1 |  |
| UBE3A | Hs00963664_g1 |  |
| MET | Hs01565582_g1 |  |
| NRXN1 | Hs00985123_m1 |  |
| HDAC1 | Hs00606262_g1 |  |
| GAPDH | Hs02786624_g1 |  |
| 18S | Hs99999901_s1 |  |
| NRN1 | sense 5'-GCTTTTCGGACTGTTTGCTC | |
|  | antisense 5'-CTGCCTTGGATGTTGAGGTT | |
